# Effect of Monosodium Glutamate on Salt and Sugar Content Reduction in Cooked Foods for the Sensory Characteristics and Consumer Acceptability

**DOI:** 10.3390/foods11162512

**Published:** 2022-08-19

**Authors:** Yehji Chung, Daeung Yu, Han Sub Kwak, Sung-Soo Park, Eui-Cheol Shin, Youngseung Lee

**Affiliations:** 1 Food R&D Planning Team, Kwang-Dong Pharmaceutical Co., Ltd., Seoul 08381, Korea; 2 Interdisciplinary Program in Senior Human-Ecology, Major in Food and Nutrition, Changwon National University, Changwon 51140, Korea; 3 Department of Food and Nutrition, Changwon National University, Changwon 51140, Korea; 4 Research Group of Food Processing, Korea Food Research Institute, Wanju 55365, Korea; 5 Department of Food Science & Nutrition, Jeju National University, Jeju 63243, Korea; 6 Department of Food Science/Institute for Food Sensory & Cognitive Science, Gyeongsang National University, Jinju 52725, Korea; 7 Department of Food Science and Nutrition, Dankook University, Cheonan 31116, Korea

**Keywords:** monosodium glutamate, salt, sodium, sugar, cooked food, rate-all-that-apply (RATA)

## Abstract

Three different types of typical Korean foods were studied to investigate the effect of monosodium glutamate (MSG) on the sensory characteristics and hedonic perception of sodium- or sugar-reduced samples. The first consumer test (*n* = 300) was conducted to evaluate the overall liking (OL) of the samples containing four different levels of salt and sugar contents without added MSG, while the second consumer test (*n* = 300) was designed to examine the effects of MSG on the samples containing reduced salt and sugar contents with the lowest observed OL in the first consumer test. The results showed that the intensity of the umami taste and saltiness of the samples increased, whereas sourness and bitterness were suppressed with added MSG. The samples with the lowest salt contents were observed to be acceptable to consumers after MSG addition, indicating a 23% reduction in sodium intake. Bitterness was partially masked, while sweetness, umami taste, and saltiness were the main factors affecting the OL after MSG addition. However, no consistent results of added MSG on the sensory attributes of samples with reduced sugar contents were observed, possibly indicating that the effect of umami taste on sweetness depends on the MSG concentration used or the type of food studied. This study could be beneficial to researchers who want to know the optimal level of MSG required to reduce the sodium or sugar contents in cooked food.

## 1. Introduction

Sodium chloride is the most widely used food additive in the food industry [1]. It serves as a flavor enhancer to improve palatability while suppressing bitterness in food [2], resulting in low consumer acceptability if the sodium content is considerably reduced [3]. Salt is also used as a preservative, texture enhancer, and food binder [4]. In addition, salt is cheaper than its other alternatives. Thus, a substantial reduction in the salt in food could be a big challenge from the standpoint of both technical and economic perspectives [3]. Excessive sodium intake is a major cause of hypertension, cardiovascular diseases, and stroke [5,6], which are diagnosed in more than 25% of the adult population worldwide [7]. Hafshajani et al. [8] reported that the average sodium intake in the world is 3950 mg/day, which is twice as high as the recommended WHO intake of 2000 mg/day. According to the Food Safety Korea (FSK) [9], sodium intake per capita in Korea was reported to be 3890 mg/day.

Several strategies have been presented to reduce the sodium and sugar contents in food. First, salt consumption can be gradually reduced by training the palate of consumers so that they adapt to less salt in their food over time. Small cumulative steps where the acceptance of the consumers for products with less sodium content can make it possible to achieve this goal, although it is time-consuming to be effectively implemented in the food industry [10]. Girgis et al. [11] reported that a 25% reduction in the sodium content of white bread could be achieved by adding salts at the time of baking with cumulative small decreases in the sodium over six weeks. Second, the salty perception is considered a multisensory characteristic, which includes not only taste, aroma, and trigeminal sensations but also texture, color, and sound [12]. Pionnier et al. [13] demonstrated an enhancement in salty perception based on salt–aroma interactions. Third, the saltiness elicited from salt can be enhanced by replacing sodium with other inorganic salts or by using natural substitutes such as mineral salts or salt enhancers [10,14].

Sugar is one of the main forms of carbohydrate, and its primary function is to provide energy to the human body. It is an essential nutrient for human tissues that use glucose as their energy source, such as the brain and nerve tissues [15]. The greatest function of sugar is related to sensory aspects; sugar imparts sweetness, which contributes to the flavor balance of food. In addition, sugar plays an important role in the Maillard reaction and freezing point and has bulking and preserving effects on food [16]. Therefore, these functional benefits of sugar prevent the food industry from reducing the sugar content in food [16]. Excessive sugar intake may cause type 2 diabetes, obesity, and cardiovascular diseases [17]. It is recommended by the WHO that sugar should represent less than 10% of the daily calorie intake [18]. Indeed, the worldwide mean sugar intake is much higher than recommended, ranging from 13.5% to 24.6% in adults and from 20.0% to 38.4% in children [19]. According to the FSK [9], the average daily sugar intake of Koreans was 76 g in 2019, which exceeded the WHO recommended standard of 50 g (based on the daily 2000 kcal requirement) and increased compared to 56 g in 2008.

One of the most widely used strategies to reduce the sugar content in food is product reformulation. The combined use of artificial sweeteners and bulking agents, such as nondigestible carbohydrates, is a good example [16]. Romagny et al. [20] reported that muffins reformulated with reduced amounts of salt, fat, and sugar could reach up to a 25% reduction in sugar content without affecting the final product quality. Another approach to reduce sugar in food is based on the multisensory characteristic, as explained for salt perception. Stieger and van de Velde [21] reported that the sweetness intensity of a food product was enhanced by the crossmodal interaction between relevant aromas and sweetness. However, this approach has been employed more for salt content reduction and is limited for sugar content reduction [21]. The degree of homogeneity of sugar distribution in food, especially solid or semisolid food, can affect the sweetness intensity. Mosca et al. [22] observed that the modulated effect of sugar distribution in agar gel was more pronounced when sugar was not homogeneously distributed in the gel for a fixed sucrose content, enhancing the sweetness intensity.

Monosodium glutamate (MSG) has been widely used as a natural flavor enhancer that elicits an umami taste [23]. According to Zanfirescu et al. [24], it is generally recognized as safe by the FAO/WHO Expert Committee on Food Additives, the U.S. Food and Drug Administration, and the European Food Safety Association. It can not only increase palatability but also reduces the amount of sodium in food [25]. The umami taste elicited by MSG affects the overall sensory profiles of the products other than simply being an enhancement in saltiness [5]. The effects of MSG on the flavor of cooked foods are broadly classified into two perspectives. First, the umami taste itself elicited by MSG can enhance the palatability and acceptability of food. Second, the synergistic effects of the umami taste with other basic tastes can lead to the reduced consumption of sodium or sugar [26]. Although studies showing the increase in perceived saltiness by MSG have been actively conducted [27], little information is available regarding the effect of MSG on the saltiness or sweetness of cooked food in terms of sensory and consumer acceptance. Therefore, this study aimed to investigate the effects of MSG on the sensory characteristics and consumer acceptance of three typical Korean foods.

## 2. Materials and Methods

### 2.1. Selection of Cooked Food

Vegetable rice porridge, soft tofu stew sauce, and simmered burdock root, representing typical home-style meals popular in Korea, were selected as cooked foods in this study. According to the FSK [9], vegetable rice porridge and soft tofu stew sauce have approximately 1400 mg and 1625 mg of sodium in one serving, based on 800 g of the porridge and 400 g of the stew sauce, respectively. They represent more than two-thirds of the recommended daily intake of sodium of 2000 mg. These two products contained many ingredients, such as vegetables, anchovies, and kelp, that elicit the umami taste; thus, it is expected that a synergistic effect of the umami taste created by the combination of various ingredients and MSG would exist. According to the FSK [9], simmered burdock root is the fifth highest in sugar content among the categories of all simmered foods in Korea and contains approximately 8.0 g of sugar in one serving (30 g).

### 2.2. Experimental Ingredients Used for Cooked Food

Refined sugar (white sugar, CJ CheilJedang Co., Seoul, Korea), refined salt (ESfood Inc., Kunpo-si, Gyeonggi-do, Korea), and MSG (Miwon, Daesang Co., Seoul, Korea) were used as raw materials commonly used for cooked foods.

#### 2.2.1. Vegetable Rice Porridge

Finely chopped Korean zucchini (160 g) and carrots (160 g) purchased in a marketplace were pan- and stir-fried in sesame oil (20 g) for 2 min and 30 s. Then, water (3200 g) and white rice (840 g) were added and boiled for 15 min. The lid was opened, and the food was stirred on a regular basis and boiled for another 30 min to completely cook the ingredients. Based on the hypothesis that the addition of MSG would enhance the saltiness [28,29], the amount of salt added to the sample was adjusted. A sample with 0.55% added salt (*w/v*) was set as the control for the preliminary experiments, and the samples with reduced salt concentrations of 10%, 20%, and 30% of the control were prepared (0.50%, 0.44%, and 0.39% salt, *w/v*, respectively). Three samples with varying amounts of MSG were prepared, showing significant decreases in the overall liking (OL) compared to the control due to the reduction in the salt content. Specifically, MSG concentrations of 0.01%, 0.04%, and 0.16% (*w/v*) were added to the samples with 0.39% salt.

#### 2.2.2. Soft Tofu Stew Sauce

After making the broth (3800 g) using dried kelp (10 slices, 5 × 5 cm) and dried anchovies (75 g), soft tofu (1050 g) and minced garlic (25 g) were added to the broth and boiled for 17 min. Finally, green onions (50 g) were added and simmered for 1 min to complete the soft tofu stew sauce. The salt concentration of the soft tofu stew sauce was determined to be 0.40% (*w/v*) as the commonly used salt concentration through the preliminary experiments. Similar to the vegetable rice porridge, a sample to which 0.40% salt was added was set as a control, and samples with reducing salt concentrations of 10%, 20%, and 30% of the control were prepared (0.36%, 0.32%, and 0.28% salt (*w/v*), respectively). Similar to the vegetable rice porridge, three different amounts of MSG of 0.01%, 0.04%, and 0.16% (*w/v*) were added to the samples for which the OL significantly decreased compared to that of the control (i.e., for the 0.28% salt sample) due to the reduction in the salt content.

#### 2.2.3. Simmered Burdock Root

The simmered burdock root was cut into 0.3 × 0.3 × 5 cm pieces and soaked in vinegar for 10 min to remove the acrid taste of burdock. The burdock (480 g) was mixed with soy sauce (120 g), sake (120 g), and water (1720 g) in a pot and boiled for 20 min with the lid open. The sugar concentration of the simmered burdock root stew was determined to be 15.0% (*w/v*) as the commonly used sugar concentration based on preliminary experiments. A sample with a 15% sugar concentration was set as a control, and samples with gradually reducing sugar concentrations of 13.5%, 10.5%, and 7.5% (*w/v*) were prepared by reducing the sugar concentration by 10%, 30%, and 50% from the control. MSG concentrations of 0.01%, 0.04%, and 0.16% (*w/v*) were added to the sample for which the sugar reduction led to a significant decrease in the OL compared with that of the control.

### 2.3. Consumer Testing

Consumer testing was conducted according to the Institutional Review Board (IRB) procedure after receiving IRB approval from Dankook University (approval number: DKU 2016-11-010). A total of 600 consumers (440 females and 160 males, aged 20–49 years) were recruited through university advertisements via flyers. They had no allergies to any food and consumed the foods tested in this study at least once a week. The consumer tests were conducted in a quiet place under incandescent lighting, and a paper ballot was used for data collection. The first group of consumers (*n* = 300) were subjected to the tests that determined the OL of the samples without the addition of MSG for each cooked food sample with varying salt concentrations. Another group of consumers (*n* = 300) participated in tests to examine the effects of MSG on salt and sugar reduction. The second consumer test was designed to examine the effects of MSG on the samples containing reduced salt and sugar contents with the least OL observed in the first consumer test. Two different consumer tests with each group (*n* = 300) were conducted a week apart.

Vegetable rice porridge and soft tofu stew sauce were served in a 70 mL paper cup with 25 g of each. The samples were kept in a thermos to maintain a temperature of 60 ± 5 °C prior to serving to the consumers. Simmered burdock root (5 g) was served to the consumers in a 70 mL paper cup. The samples were kept in a thermos to maintain a temperature of 5 ± 1 °C prior to serving. The consumer tests were performed in a sequential monadic method with the Williams design to balance sample presentation orders for each serving position. The samples were provided in paper cups labeled with three-digit random codes. Spring water and unsalted crackers were provided to consumers for palate cleansing between the samples. Consumers were first asked to rate the OL of each sample using the nine-point verbal hedonic scale (1 = extreme dislike to 9 = extreme like), followed by a RATA questionnaire consisting of five basic tastes (sweet, salty, sour, bitter, and umami) in common and additional flavor attributes, depending on the samples evaluated. For the RATA method, consumers were asked to check the attributes they thought were appropriate for describing the samples and then to rate the intensity of the checked terms based on a three-point intensity scale (“low,” “medium,” and “high”). The attribute orders for the RATA questionnaire were randomized among consumers. Since a three-point intensity scale was used with the RATA question, participants were given a short orientation on how to use the RATA question attached with scales. The attributes of each food were determined based on preliminary works in a laboratory, and a short orientation on how to evaluate the attributes was provided prior to the test. The preliminary works to identify the attributes were conducted by descriptive panelists and finalized on their consensus. Definitions of the attributes were given on the request of participants.

### 2.4. Sodium and Sugar Content Analysis

The sodium content was analyzed according to Seo et al. [30]. First, 10 mL of nitric acid was added to 0.3 g of the sample and left for 1 h, followed by decomposition using a microwave (Ethos Easy, Milestone, Fatebenefratelli, Sorisole, Italy). The decomposition solution was allowed to cool, transferred to a 100 mL volumetric flask, and used as a test solution after distillation. The standard solution was 0.5 M nitric acid. The prepared test solution and standard solution were analyzed by ICP-OES (Optima 8300, Perkin Elmer, Waltham, MA, USA).

The sugar content was analyzed, with a slight modification, according to Park et al. [31]. After homogenizing the sample, fat was removed from the sample and weighed; subsequently, the separated fat was added to 25 mL of distilled water. This mixture was heated for 25 min in a water bath at 85 °C to extract the saccharides, cooled to room temperature, filtered with a 0.45 μm nylon membrane filter, and injected with 10 μL for use as a test solution. At this time, when the test solution was turbid, it was centrifuged at 2000 rpm for 10 min, filtered, and analyzed using HPLC (Agilent 1100, Agilent, Santa Clara, CA, USA). Fructose, glucose, sucrose, maltose, and lactose standards were taken, dissolved in distilled water, diluted, and used as a standard solution for preparing a calibration curve.

### 2.5. Statistical Analysis

The OL scores were analyzed using a two-way ANOVA, treating the samples as a fixed effect of variation and the consumers as a random effect. The OL means were separated using a least significant difference to determine whether there was a significant difference between the samples (α = 0.05). The results obtained from the RATA method were analyzed by designating the unselected attributes as 0 and designating the frequencies as low—1, medium—2, and high—3 for the selected attributes [32]. Significant differences in the summed frequencies of the sensory attributes for each sample obtained from the RATA method were analyzed by Cochran’s Q test. Partial weighted regression coefficients were calculated using a partial least squares (PLS) regression to numerically express the relationship between the RATA attributes (X matrix: independent variable) and OL (Y matrix: dependent variable). Although there has been an issue with treating the frequency data obtained using the RATA method as continuous data, this type of data was successfully implemented as numerical values [33,34,35,36]. Data analyses were performed using SAS (version 9.1, SAS Inst. Inc., Cary, NC, USA) and XLSTAT (version 2017, Addinsoft, Paris, France).

## 3. Results and Discussion

### 3.1. The OL of Vegetable Rice Porridge and Soft Tofu Stew Sauce According to the Concentrations of Only Salt and Salt with MSG

Table 1 shows the results of the consumer OL and sodium contents of the vegetable rice porridge and soft tofu stew sauce containing various concentrations of only salt and salt with MSG. For the vegetable rice porridge, the OL of the samples with 0.39% salt was significantly lower than that of the control (0.55% salt) in the absence of MSG. However, when a 0.16% concentration of MSG was added to the samples with 0.39% salt, the OL of the samples was significantly higher than that of the control. The sodium content of the samples with 0.39% salt was 1752.2 mg/kg, showing an approximately 22.9% sodium reduction with higher OL compared to that of the control (2271.5 mg/kg).

Similar results were found for the soft tofu stew sauce (Table 1). Samples containing the lowest salt content (0.28%) showed a significantly lower OL compared to that of the control (0.4% salt). When a 0.16% concentration of MSG was added to the samples with 0.28% salt, the OL drastically increased and was higher than that of the control. The samples with 0.28% salt and 0.16% MSG (Na: 1681.9 mg/kg) were better liked by the consumers, even with a salt reduction of 13.7% compared to the control (Na: 1949.8 mg/kg). This observation was consistent with the study conducted by Maheshwari et al. [37], which reported that the addition of MSG improved the OL of traditional Indian bread when compared with that of control prepared without MSG.

A decrease in the palatability of a sample due to a reduction in the salt content generally occurs in most foods, mainly due to a decrease in the overall flavor of the food with a decrease in saltiness [38,39]. Saltiness reportedly improves the overall flavor of food by improving the balance with other basic tastes such as sweetness and sourness [38,39]. The property of MSG to enhance flavor and increase the level of perceived saltiness has been validated [27,40]. MSG contains two-thirds less sodium compared to salt [26], which would enable MSG to reduce the amount of salt added to food. The effects of a small amount of added MSG in soups or other types of food to reduce added salts have already been investigated by many studies [41]. Okiyama and Beauchamp [28], and Roininen et al. [29] also reported that the increase in the palatability of foods due to added MSG is because of the synergistic effect of the umami taste elicited from MSG, which strengthens the salty and savory flavor with increased balance with the other tastes.

Table 2 shows the consumer OL and sugar content of simmered burdock root for varying concentrations of only sugar and sugar with MSG. The OL of the samples not containing MSG gradually decreased with the decrease in the sugar content, indicating that sweetness elicited from sugar plays a major role in dictating the consumer OL of the simmered burdock root. Sweetness has been known as a driver of liking most sugar-based foods [42,43]. The sugar content of the samples also decreased from 145.7 g/kg for the control (15% sugar) to 90.2 g/kg for the sample (7.5% sugar). In contrast with the salt-based samples, when MSG was added to the sample with the lowest OL (7.5% sugar), no improvement in the OL was observed for the samples. The OL of samples with added MSG was rather lowered than that of samples without added MSG. This suggests that the effect of MSG on the reduction in the sugar content in this type of food may be minimal. This may indicate that supplemented umami taste led to the breakdown of flavor balances of this sample. This would be more discussed hereafter.

Conversely, Fuke and Ueda [44] reported that the umami taste elicited from low concentrations of nucleic acid components such as AMP, which is a form of phosphoric acid binding to ribose, increased the sweetness. The effect of the umami taste on the sweetness may be dependent on the umami intensity, and this should be investigated in future research.

### 3.2. RATA Counts of Samples According to the Concentrations of Only Salt and Salt with MSG or Only Sugar and Sugar with MSG

Table 3 shows the frequencies of the sensory characteristics obtained by using the RATA method for vegetable rice porridge prepared with only salt and salt with MSG. As shown in Table 3, the saltiness and umami taste significantly decreased, as the salt concentrations decreased, whereas the bitterness tended to increase. As the salt content decreased, the masking effect of saltiness on bitterness is believed to decrease [45,46], showing that consumers perceived the bitterness of the product more strongly. This observation was in line with results of Breslin and Beauchamp [47], who reported that salts such as NaCl, LiCl, and KCl could suppress bitterness by up to 55%, although this suppression was not equivalent to the reciprocal suppression of saltiness. They pointed out that the key component in this effect was the sodium or lithium ion that showed a suppressive effect on bitterness. In addition, the umami taste significantly decreased in the sample with a salt concentration of 0.39%. Although no MSG was added to the sample with a salt concentration of 0.55%, it showed the highest RATA counts in terms of the umami taste. Vegetables can be a good source of potential umami taste due to IMP and glutamic acid that are naturally present in vegetables [48,49]. We believe that zucchini and carrots used for cooking the vegetable rice porridge provided additional sources of the umami taste.

When MSG of 0.16% was added to the vegetable rice porridge, saltiness and umami taste were the two attributes that significantly increased (Table 3). The RATA counts in saltiness was considerably increased from 79 for the sample containing 0.39% salt only to 155 for the sample containing 0.39% salt and 0.16% MSG. The RATA counts in the umami taste of the sample containing 0.39% salt and 0.16% MSG was significantly higher than that of the control. The increase in the saltiness of the food with added MSG has been reported in many studies [50,51]. The saltiness of MSG is reportedly associated with the G-protein-coupled receptor, which enhances the perception of saltiness [52]. Chae et al. [53] reported that when 10% sodium inosinate was mixed with MSG, the mixture was 17 times saltier than sodium guanylate. The enhanced effect of MSG on the increased saltiness and palatability of chicken broth has also been studied [54]. MSG added to food can promote saliva secretion and enhance the intensity and persistence of the flavor of food while playing an important role in improving palatability [49].

Table 4 presents the RATA counts for the soft tofu stew sauce samples containing only salt and salt with MSG. As observed for the vegetable rice porridge, the saltiness of the samples containing only salt significantly decreased as the salt concentration decreased. However, no differences were observed for the remaining characteristics, including the umami taste, even for the lowest salt content (0.28%). The same trend was observed for the samples containing MSG. It is interesting to note the different results of the umami trends between the vegetable rice porridge and soft tofu stew sauce. As opposed to the vegetable rice porridge, the soft tofu stew sauce contains ingredients such as kelp and anchovies that generate a strong umami taste. This might prevent the umami taste in the soft tofu stew sauce from being reduced, even for the lowest salt content (0.28%). Another possible reason is the different viscosities of the two samples. Im et al. [55] reported that the sensory characteristics of the soft tofu stew sauce seemed to be less perceived for more viscous samples. In this study, the viscosity of the soft tofu stew sauce was higher than that of the vegetable rice porridge (data not shown). According to Roberts et al. [56], thickeners such as guar gum allow consumers to taste less flavor because they combine with the structure of food to hold the volatile components of the food.

Table 4 interestingly shows that the sweetness of the soft tofu stew sauce increased as the MSG content increased. The sample containing 0.16% MSG and 0.28% salt had a higher sweetness by 1.5 times compared to that of the control (i.e., RATA counts from 20 to 30), but it was not significant. The interaction between the umami taste and saltiness has not yet been fully clarified, although there are numerous studies that deal with the binary interaction between the umami taste and other basic tastes [57,58,59]. Only a few studies focusing on the umami–sweetness interaction in aqueous solutions have been published, but very few are related to cooked foods. Koo [60] reported that sweetness decreased when 0.8% MSG was mixed with 10% sugar in an aqueous solution model system. In contrast, Fuke and Ueda [44] reported that the umami taste elicited from a low concentration of a nucleic acid component increased sweetness. Heyer et al. [61] reported that the umami taste could tune sweetness-like characteristics because MSG, natural sugars, and artificial sweeteners have the convergence of afferent signals.

Table 5 shows the RATA counts for samples with only sugar and sugar with MSG for simmered burdock root. The sweetness and umami taste of the samples significantly decreased as the sugar content decreased. However, no clear trend was observed for added MSG with a 50% reduction in sugar concentration. As mentioned earlier, the effect of MSG on sweetness has not yet been clarified, and the same consequence was observed in this study. Yamaguchi [49] and Halpern [62] reported that the effect of MSG on the preference of foods varies depending on the type of food.

### 3.3. Identification of the Drivers for the OL of Samples with and without MSG

**Figure 1:**
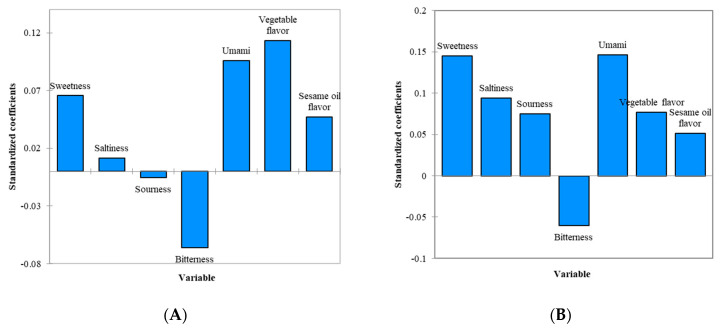
(**A**) Standardized coefficients from the PLS regression for vegetable rice porridge with 0.55% salt in the absence of MSG. (**B**) Standardized coefficients from the PLS regression for vegetable rice porridge with 0.39% salt in the presence of 0.16% MSG.

**Figure 2:**
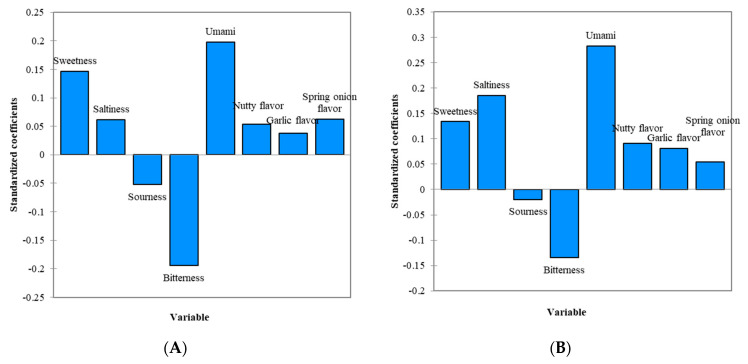
(**A**) Standardized coefficients from the PLS regression for soft tofu stew sauce with 0.40% salt in the absence of MSG. (**B**) Standardized coefficients from the PLS regression for soft tofu stew sauce with 0.28% salt in the presence of 0.16% MSG.

**Figure 3:**
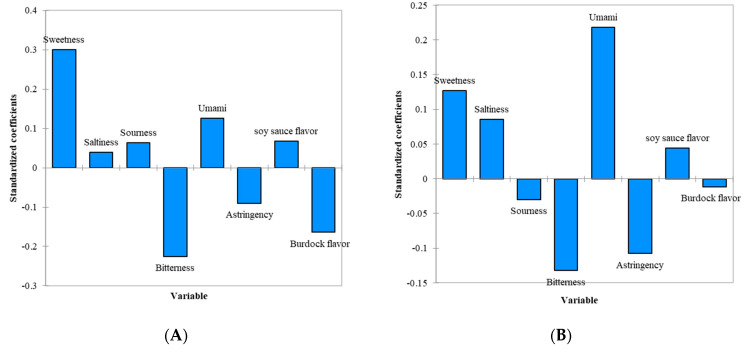
(**A**) Standardized coefficients from the PLS regression for simmered burdock root with 15.0% sugar in the absence of MSG. (**B**) Standardized coefficients from the PLS regression for simmered burdock root with 7.5% sugar in the presence of 0.16% MSG.

Weighted regression coefficients for a PLS regression predicting the OL of the samples from the sensory characteristics obtained from the RATA counts are graphically presented in Figure 1, Figure 2 and Figure 3. These sensory characteristics were weighed by their standard deviations to obtain and compare the relative importance of the characteristics to the OL [63]. Figure 1 shows the standardized coefficients from the PLS regression for the vegetable rice porridge containing 0.55% salt only and 0.39% salt with 0.16% MSG. Sweetness, umami taste, vegetable flavor, and sesame oil flavor were identified as the key positive sensory characteristics driving the OL, whereas bitterness was found to have a negative effect on the OL, indicating that consumers liked this sample when it was less bitter (Figure 1A). For the sample containing 0.39% salt with 0.16% MSG, sweetness, umami taste, saltiness, sourness, and vegetable flavors were found to be the main factors affecting the OL, and all of the main factors were positive drivers of the OL (Figure 1B). Increases in the standardized coefficients for sweetness, saltiness, and sourness and a decrease in the standardized coefficient for bitterness were pronounced after 0.16% MSG was added. This indicates that bitterness detected in the vegetable rice porridge could be partially masked by added MSG, as discussed earlier [64]. It is interesting to note that besides the enhancement of saltiness, the contribution of sweetness and sourness as liking drivers also increased with added MSG. This is because a balanced flavor including the sweetness and sourness of the vegetable rice porridge was achieved by enhanced saltiness due to the added MSG [38,39]. Overall, it is thought that consumers’ palatability and the flavor balances of this sample could be enhanced due to the added MSG [28].

Figure 2 presents the standardized coefficients obtained from the PLS regression for the soft tofu stew sauce containing 0.40% salt only and 0.28% salt with 0.16% MSG. While the umami taste and sweetness were the most important positive characteristics driving the OL, bitterness was identified as a factor that negatively drives OL (Figure 2A). Similar to the vegetable rice porridge, sweetness, umami taste, and saltiness were found to be the main factors affecting the OL when 0.16% MSG was added (Figure 2B). The contribution of bitterness to the OL also decreased after the addition of MSG, indicating that the consumers liked this sample when it was less bitter. Unlike the vegetable rice porridge, sweetness contributed less to the OL of this sample after the addition of MSG. This is probably due to unclear complexity between the tastes of sweetness and umami [44,60,61], as discussed earlier.

Figure 3 shows the standardized coefficients obtained from the PLS regression for the simmered burdock root containing 15% sugar only and 7.5% sugar with 0.16% MSG. Sweetness, bitterness, and burdock flavor were found to be the main factors affecting OL, among which only sweetness had a positive effect on the OL, while bitterness and burdock flavor were negative drivers (Figure 3A). After the addition of MSG, the umami taste, bitterness, and sweetness were found to be the main factors affecting the OL, showing that the umami taste and sweetness were positive drivers, while bitterness was a negative driver for the OL. With the addition of MSG, the contribution of sweetness to the OL decreased, and the umami taste increased considerably. Moreover, astringency contributed more negatively to the OL of this sample. We believed that added MSG might cause the overall flavor of this sample to be unbalanced, lowering the OL of this sample. This was also observed in Table 2, showing that the samples with added MSG were less liked than the samples without added MSG. Hong et al. [65] reported that MSG would have different tastes depending on the concentration used in a solution or food. It could be mostly salty, greasy, sweet, and umami at low concentrations, while a bitter or nauseous taste could be described at high concentrations.

## 4. Conclusions

Although taste–taste interactions are not fully understood, a number of studies have been conducted to elucidate the interactions between different basic tastes. However, this has not been satisfactorily extended to cooked food, possibly due to the complicated interactions such as the enhancement or suppression of the mixture of tastes and odors present in the food. In this view, the findings of this study could be meaningful for examining the interaction between umami elicited from MSG and other basic tastes in cooked foods, although the results should be limited to the three typical Korean foods used in this study.

The results showed that the samples in this study with the lowest salt contents and added MSG were acceptable to consumers, indicating that an ~23% reduction in the sodium content with higher consumer acceptability could be achieved. However, no significant effect of MSG on the reduction in the sugar content was observed for the simmered burdock root, indicating that the effect of the umami taste on sweetness may depend on the MSG concentration used or the type of food studied.

As opposed to salt and sugar, which have a critical level for their application in food from a standpoint of health according to the FAO or WHO, no ADI (acceptable daily intake) is established for MSG, to the authors’ best knowledge, according to the FAO/WHO because it is safe to be used in any type of food. From this point of view, therefore, the authors believe that if the excessive intake of salt or sugar can be partially replaced with MSG without any negative changes in food quality, it could be nice to reduce any potential risks occurring from the excessive intake of salt or sugar. Future works should be extended to other types of foods, including commercial foods, to comprehensively examine the effects of MSG on salt and sugar content reduction in foods. Specifically, more studies are needed to examine the effects of MSG on the sweetness of foods.

## Figures and Tables

**Table 1:** Consumer overall liking and sodium contents of vegetable rice porridge and soft tofu stew sauce containing various concentrations of salt or salt with MSG.

Foods	Refined Salt (%)	MSG (%)	OL ^1^	Sodium Contents (mg/kg)
Vegetable rice porridge	0.55	0	6.0 a ^2^	2271.5 ± 1.6 ^3^
	0.50	0	5.9 a	2018.4 ± 12.1
	0.44	0	5.5 ab	1804.5 ± 14.0
	0.39	0	5.0 b	1595.3 ± 19.7
	0.55	0	5.4 b	2271.5 ± 1.6
	0.39	0.01	5.4 b	1594.1 ± 37.2
	0.39	0.04	5.6 b	1638.8 ± 9.1
	0.39	0.16	6.6 a	1752.2 ± 47.3
Soft tofu stew sauce	0.40	0	5.4 a	1949.8 ± 3.4
	0.36	0	5.1 a	1764.4 ± 15.2
	0.32	0	5.1 ab	1637.7 ± 11.9
	0.28	0	5.0 b	1474.5 ± 12.8
	0.40	0	5.5 b	1949.8 ± 3.4
	0.28	0.01	4.8 b	1483.9 ± 4.1
	0.28	0.04	5.2 b	1530.1 ± 15.7
	0.28	0.16	5.8 a	1681.9 ± 4.6

^1^ Overall liking. ^2^ Means with different letters in the same column for each food are significantly different (*p* < 0.05). ^3^ Mean ± standard deviation.

**Table 2:** Consumer overall liking and sugar contents of simmered burdock root containing various concentrations of sugar or sugar with MSG.

Food	Sugar (%)	MSG (%)	OL ^1^	Sugar Contents (g/kg)
Simmered burdock root	15.0	0	5.9 ab ^2^	145.7 ± 3.3 ^3^
	13.5	0	6.1 a	130.2 ± 12.2
	10.5	0	5.4 bc	101.8 ± 4.7
	7.5	0	4.9 c	90.2 ± 1.5
	15.0	0	5.9 a ^2^	145.7 ± 3.3
	7.5	0.01	4.9 b	93.6 ± 10.1
	7.5	0.04	5.0 b	83.7 ± 6.5
	7.5	0.16	5.1 b	97.3 ± 5.3

^1^ Overall liking. ^2^ Means with different letters in the same column for each case are significantly different (*p* < 0.05). ^3^ Mean ± standard deviation.

**Table 3:** RATA counts according to salt or salts with MSG concentrations for vegetable rice porridge.

		Sweetness	Saltiness	Sourness	Bitterness	Umami	Vegetable	Sesame Oil
Refined salt (%)	0.55	27 a ^1^	200 a	6 a	7 ab	11 9 a	121 a	164 a
	0.50	30 a	140 b	5 a	4 b	122 a	141 a	144 ab
	0.44	26 a	108 c	7 a	8 ab	92 b	142 a	136 b
	0.39	21 a	79 d	4 a	16 a	80 b	145 a	137 b
Refined salt + MSG (%)	0.55	41 a ^1^	181 a	6 a	9 a	112 b	117 a	116 a
	0.39 + 0.01	27 a	74 c	6 a	5 a	97 b	125 a	121 a
	0.39 + 0.04	38 a	95 c	7 a	9 a	98 b	133 a	113 a
	0.39 + 0.16	41 a	155 b	4 a	6 a	152 a	116 a	127 a

^1^ Values with different letters in the same column for each case are significantly different according to Cochran’s Q test (*p* < 0.05).

**Table 4:** RATA counts according to salt or salt with MSG concentrations for soft tofu stew sauce.

		Sweetness	Saltiness	Sourness	Bitterness	Umami	Nutty Flavor	Garlic	Spring Onion
Refined salt (%)	0.40	23 a ^1^	93 a	12 a	22 a	79 a	34 a	55 a	50 a
	0.36	20 a	89 a	11 a	27 a	73 a	30 a	53 a	49 a
	0.32	23 a	87 ab	9 a	23 a	78 a	30 a	60 a	44 a
	0.28	30 a	76 b	12 a	18 a	76 a	48 a	53 a	41 a
Refined salt + MSG (%)	0.40	20 ab ^1^	86 a	7 a	25 a	72 a	34 a	71 a	66 a
	0.28 + 0.01	18 b	66 c	10 a	21 a	68 a	33 a	68 a	68 a
	0.28 + 0.04	23 ab	73 bc	5 a	20 a	74 a	36 a	63 a	65 a
	0.28 + 0.16	30 a	80 ab	9 a	14 a	75 a	42 a	63 a	65 a

^1^ Values with different letters in the same column for each case are significantly different according to Cochran’s Q test (*p* < 0.05).

**Table 5:** RATA counts according to sugar or sugar with MSG concentrations for simmered burdock root.

		Sweetness	Saltiness	Sourness	Bitterness	Umami	Astringency	Soy	Burdock
Sugar (%)	15.0	192 a ^1^	166 a	25 a	12 a	110 a	23 a	181 a	171 a
	13.5	165 a	173 a	25 a	10 a	123 a	24 a	182 a	174 a
	10.5	132 b	163 a	18 a	18 a	97 ab	27 a	180 a	159 a
	7.5	89 c	188 a	30 a	13 a	79 b	26 a	184 a	172 a
Sugar + MSG (%)	15.0	184 a ^1^	162 b	16 ab	14 b	102 a	24 b	161 b	156 a
	7.5 + 0.01	97 b	215 a	17 ab	32 ab	85 a	53 a	197 a	145 a
	7.5 + 0.04	77 b	222 a	31 a	44 a	83 a	56 a	184 ab	143 a
	7.5 + 0.16	101 b	222 a	13 b	26 ab	84 a	53 a	197 a	139 a

^1^ Values with different letters in the same column for each case are significantly different according to Cochran’s Q test (*p* < 0.05).
